# A170 A RARE CASE OF CELIAC DISEASE WITH PULMONARY HEMOSIDEROSIS AND DILATED CARDIOMYOPATHY

**DOI:** 10.1093/jcag/gwad061.170

**Published:** 2024-02-14

**Authors:** E K Dessie

**Affiliations:** Pediatrics and child health, Sick Kids Foundation, Toronto, ON, Canada

## Abstract

**Background:**

Celiac disease (CD) is a primary disease of the intestine but patients also present with extra intestinal manifestations like iron deficiency anemia, dermatitis herpetiformis, selective IgA deficiency, thyroid disorders, diabetes mellitus and various connective tissue disorders but rarely with pulmonary hemosiderosis and dilated cardiomyopathy.

**Aims:**

We present a rare case of CD associated with pulmonary hemosiderosis and dilated cardiomyopathy in a 10 year old child.

**Methods:**

Patient interview and chart review was conducted. Literature reveiw was performed using pub med data base.

**Results:**

A 10 year old presented with a 1 day of cough and hemoptysis. His past medical history includes Constipation and low percentile for weight and height. His first cousin is recently diagnosed with celiac disease, otherwise his family history is unremarkable. He is pale on exam and has a BMI of 13.64 which is ampersand:003C1% for age. He had fine crackles on bilateral lung fields but no signs of respiratory distress. His pericardium was active but no murmur or gallop. CXR showed cardiomegaly and Echo was suggestive of dilated cardiomyopathy. ECG was significant for LBBB. Chest CT showed bilateral confluent symmetric ground glass densities with possibility of pulmonary hemosiderosis. His blood work was significant for iron deficiency anemia, low selenium of 0.94umol/L and zinc 8.4umol/L. His antibody screen was positive for ANA, a tTGA of ampersand:003E4,965.5 and positive EMA. The cardiomyopathy and pulmonary hemosiderosis in this case are considered to be secondary findings as both have been reported in the context of celiac disease and patient was initiated on gluten free diet (GFD). Patient was blood transfused and kept on oral iron and multivitamins. He was also started on aldactazide 1 tab daily, 20mg twice daily enteresto and carvedilol 3.125mg twice daily as part of his heart failure management. At one month follow up, patient was having abdominal discomfort due to his constipation and follow up labs showed improvement of his anemia.

**Conclusions:**

Patients with pulmonary hemosiderosis occuring with CD demonstrate improvement of their symptoms with GFD highlighting the importance of screening all pulmonary hemosiderosis patients for CD. Cardiomyopathy associated with celiac disease is also another very rare occurrence and only few studies have been reported so far.

In conclusion, although a rare occurrence, the association of CD with pulmonary hemosiderosis and cardiomyopathy has huge therapeutic implications emphasizing the need to have high index of suspicion and routine screening for CD among patients with pulmonary hemosiderosis and dilated cardiomyopathy.

**Funding Agencies:**

The hospital for sick children/sick kids hospital

